# Biomimetic inorganic camouflage circumvents antibody-dependent enhancement of infection†
†Electronic supplementary information (ESI) available. See DOI: 10.1039/c7sc03868b


**DOI:** 10.1039/c7sc03868b

**Published:** 2017-10-20

**Authors:** Xiaoyu Wang, Yong-Qiang Deng, Dong Yang, Yun Xiao, Hui Zhao, Qing-Gong Nian, Xurong Xu, Xiao-Feng Li, Ruikang Tang, Cheng-Feng Qin

**Affiliations:** a Qiushi Academy for Advanced Studies , Zhejiang University , Hangzhou , Zhejiang 310027 , China . Email: rtang@zju.edu.cn; b Department of Virology , State Key Laboratory of Pathogen and Biosecurity , Beijing Institute of Microbiology and Epidemiology , Beijing , 100071 , China . Email: qincf@bmi.ac.cn; c Centre for Biomaterials and Biopathways , Department of Chemistry , Zhejiang University , Hangzhou , Zhejiang 310027 , China

## Abstract

Pre-existing antibodies can aggravate disease during subsequent infection or vaccination *via* the mechanism of antibody-dependent enhancement (ADE) of infection. Herein, using dengue virus (DENV) as a model, we present a versatile surface-camouflage strategy to obtain a virus core-calcium phosphate shell hybrid by self-templated biomineralization. The shelled DENV stealthily avoids recognition by pre-existing antibodies under extracellular conditions, resulting in the efficient abrogation of the ADE of infection both *in vitro* and *in vivo*. Moreover, the nanoshell can spontaneously degrade under intracellular conditions to restore the virus activity and immunogenicity due to its pH-sensitive behaviour. This work demonstrates that the biomimetic material shell can significantly improve the administration safety and potency of the DENV vaccine, which provides the promising prospect of chemically designed virus-material hybrids for immune evasion.

## Abstract 2

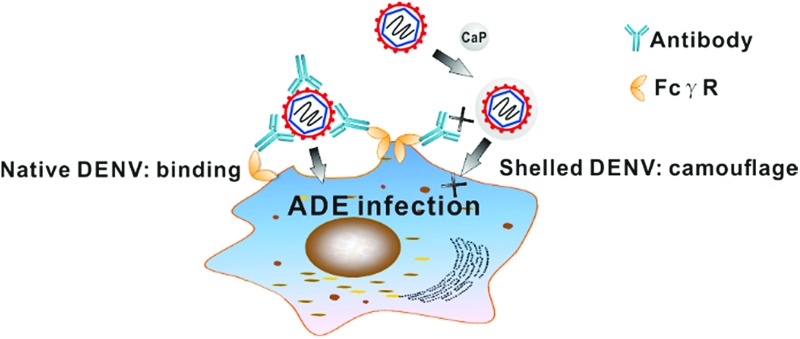
A biomimetic surface camouflage is applied to abrogate antibody-dependent enhancement of DENV infection, providing a flexible tactic for immune evasion.

## Introduction

The efficacy of current biomedicine is highly restricted by unwanted immune responses.^1^–^3^ In nature, to hide from immune surveillance, a few pathogenic bacteria use host serum albumin to form a stealth surface coating.^4^ Inspired by such a phenomenon, nanoparticles that are coated with endogenous cell components can provide a stealth effect to overcome *in vivo* immune clearance without compromising the activity.^5^–^7^ Although previous attempts have verified that the coating of instinct components onto nanoparticles could confer an *in vivo* immune evasion effect, biomimetic camouflage strategies remain rarely mentioned. We note that some living organisms have the ability to produce biomineral shells, which are biocompatible and endow protective functions.^8^ Inspired by the unique characteristics of biominerals, we propose the incorporation of a biomimetic mineral shell onto the outside of a virus could facilitate the camouflage effect to circumvent pre-existing immunity.

Pre-existing antibodies can increase the severity of viral infectious diseases in humans upon secondary infection or administration, which is designated as antibody-dependent enhancement (ADE) of infection.^9^ This effect is critically serious in dengue virus (DENV) infection since 390 million cases per year generate a high baseline of pre-existing anti-DENV antibodies among people worldwide, leading to the risk of fatal ADE of infection due to secondary exploration.^10^ Notably, the newly approved dengue vaccine is not recommended for use in children under nine years old due to the risk of ADE.^11^–^13^ During ADE of infection, a pre-existing antibody recognizes the virus and forms a virus–antibody complex to promote the entry and replication of DENV through ligation of the antibody Fc portion to Fcγ receptors on monocytes.^14^,^15^ Theoretically, viral epitopes that bind with pre-existing antibodies can be subtracted or blocked to abrogate the ADE of infection.^16^ However, the modification of virus surfaces without compromising the native activity of the virus still remains a great challenge.^17^ Chemical camouflage is advantageous for virus camouflage owing to its flexibility and low-cost.

A key challenge of forming an optimal camouflage is that the coating should be unrecognized by antibodies and be switchable: evading the undesired antibodies under extracellular conditions, meanwhile precisely degrading to ensure the original bioactivity under intracellular conditions. It is noteworthy that abundant endogenous calcium phosphate (CaP) phases are naturally formed in human intestines as a biological self-component; they can avoid body clearance and chaperone antigens to intestinal immune cells.^18^ Moreover, negatively charged amino residues on viral surfaces benefit the *in situ* biomineralization, and the formed CaP shell could afford pH sensitive biodegradation under endosomal pH conditions.^19^–^21^ Thus, the incorporation of such bio-originated biominerals to viral surfaces could meet the needs for the proposed immune camouflage. In the present study, by using the self-templated biomineralization of DENV, we report that viral particles can be contained within a biodegradable CaP shell, and the resulting DENV-CaP core–shell hybrids can circumvent the ADE of infection as well as maintain the original immunogenicity of DENV.

## Results and discussion

### DENV-templated biomineralization

**Fig. 1:**
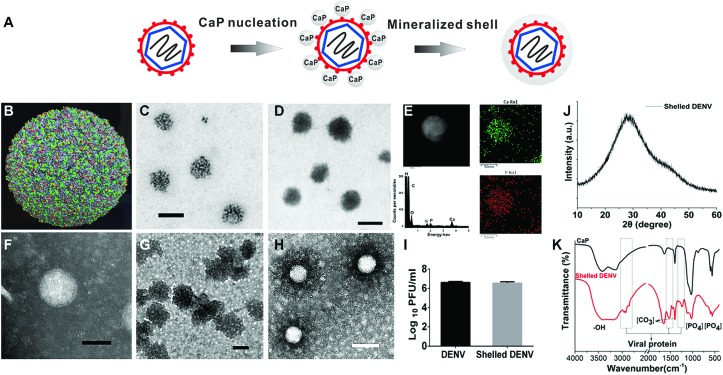
Physiochemical characters of shelled DENV. (A) The scheme illustrates that viral surfaces can initiate the formation of calcium phosphate nanoshells. (B) In the resolved cryo-electron microscopy (Cryo-EM) structure of a dengue virion, the glutamic acid and aspartic acid that are exposed on the virus surface are coloured in green and red using PyMol software (Protein Data Bank: 3J27). (C) Non-stained TEM showing viral surface directed the nucleation of CaP nanoclusters at an initial stage of biomineralization (scale bars: 100 nm). (D) Non-stained TEM showed the biomineralized DENV nanoparticle (scale bars: 100 nm). (E) Scanning TEM image of a single shelled DENV particle (scale bar: 50 nm). *In situ* elemental mapping of the nanoparticle revealed the presence of Ca and P on the surface of the nanoparticle. EDS spectra showed the surface elementary composition. The Si signal was caused by an accessory for the instrument. (F) Phosphotungstic acid stained DENV, scale bar: 50 nm. The grey background indicated the grid was covered with a layer of heavy metal salts (phosphotungstic acid). (G) The negatively stained pure CaP nanoparticles showed a solid sphere structure, indicating no biological components, scale bar: 100 nm. (H) Negatively stained shelled DENV exhibited a spherical virus-core and CaP-shell structure. The hollow structures of single particles revealed viral particles had a low electron density and cannot be stained. The particle size is identical to the original virus. The area surrounding the white core is darker than the background, indicating a higher electron density than the phosphotungstic acid layer because the CaP shell increased the electron density surrounding the virus core. (scale bars: 100 nm). (I) Identification of the enclosed DENV from the hybrid by plaque formation assay. (J) XRD analysis of shelled DENV. (K) FT-IR analysis of shelled DENV.

The approach for DENV-directed mineralization was feasibly achieved by adding CaCl_2_ to sodium phosphate monobasic containing Dulbecco’s modified Eagle medium (DMEM) supplemented with DENV particles (Fig. 1A). The abundant glutamic acid (green) and aspartic acid (red) displayed on the outside of the DENV nanoparticles aid in concentrating calcium ions and triggering the *in situ* nucleation (Fig. 1B).^22^ Zeta potential measurement showed that native DENV had a surface charge of –15.6 mV at pH 7.4 due to its anionic carboxylate groups. At an early stage of mineral deposition, we found the electron dense nanoclusters spontaneously assembled outside the viral particle, indicating the presentation of acidic amino acids promoting the mineral nucleation on the viral surface, as evidenced by non-stained transmission electron microscopy (TEM, Fig. 1C). The nano hybrid was identified by the coexistence of the CaP mineral phase and virus by energy dispersive X-ray spectroscopy (EDS) and Fourier transform infrared spectroscopy (FT-IR, Fig. S2B,† 1J and K). These results indicated that viral surfaces can efficiently induce a heterogeneous CaP deposition. Such DENV-templated mineralization was consistent with the previous understanding that the negatively charged biomolecules can induce biomineral nucleation.^23^,^24^

The prolonged deposition of CaP on the viral surface further produced nanoparticles with a greater electron density (shelled DENV) than the bare ones (Fig. 1D and F). To ensure the presence of the inner viral core, the hybrid was negatively stained. The result depicted hollow spheres contained a white core with an average diameter of around 60 nm, this corresponded to the native virus particle (Fig. 1F and H), because the viral particle has a low electron density and cannot be stained. In contrast, the staining treatment of CaP nanoparticles exhibited a solid sphere structure with an increased electron density compared with that of the phosphotungstic acid background, indicating the enhanced electron density of CaP in the staining TEM (Fig. 1G). In Fig. 1H, the area surrounding the white core is darker than the background, representing a CaP shell. The negative staining TEM revealed a distinct core–shell structure as compared with the pure CaP control. To further confirm the formation of a CaP shell on a single nanoparticle, we used scanning transmission electron microscopy (STEM) to investigate the coexistence of Ca and P elements on shelled DENV (Fig. S3†). The energy dispersive X-ray spectroscopy (EDS) attached to the STEM confirmed that Ca and P are distributed on the exterior of the shelled DENV (Fig. 1E), indicating that the shelled DENV was similar to endogenous CaP both in phase and size. The virus enclosed in the complex was confirmed by the infectivity of the shelled DENV (Fig. 1I). This material-based modification strategy could be generally applied to all four serotypes of DENV with high efficiency (Fig. S1 and S2†).

### Reversible bioactivity of camouflaged DENV

**Fig. 2:**
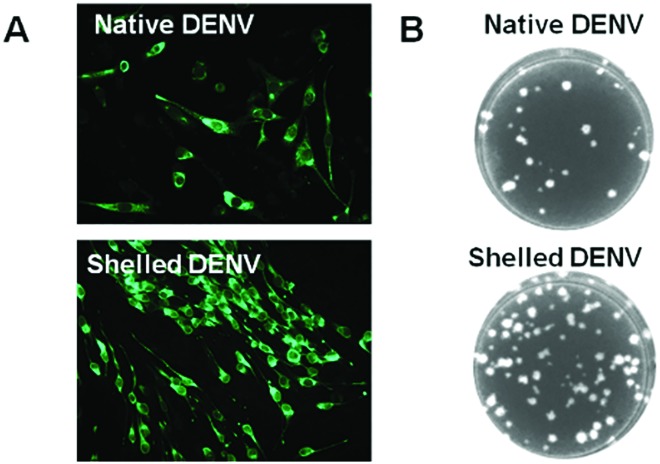
Shelled-DENV is biodegradable to restore viral bioactivity. (A) The expression of the viral envelope protein was detected by DENV-specific antibody 2A10G6 at 72 h post infection. (B) The infectivity of the shelled DENV was examined by a plaque formation assay at 72 h post infection.

In order to investigate whether the biomimetic CaP shell is biodegradable, to release the enclosed virus, and whether the released virus remains active, we examined the capability of the shelled virus to translate and replicate the envelope (E) protein. The indirect immunofluorescence-staining assay (IFA) showed that the viral E protein was expressed efficiently in BHK21 cells after incubating with shelled DENV (Fig. 2A and S4†). Furthermore, the plaque formation assay revealed that the shelled DENV (81 plaques) is a little more infectious than the native DENV (33 plaques) (Fig. 2B and S4†). Such phenomena could be explained by the well-established understanding about the receptor-independent cellular uptake pathway of CaP nanoparticles and the pH-switchable degradability of CaP within intracellular microenvironments, which enhances the delivery of virion to BHK-21 cells, resulting in the increasing number of plaques formed.^25^ Additionally, the CaP nanoshell exhibited no extra cytotoxic effect on the cells compared with the native DENV due to its similarity to endogenous materials (Fig. S5†).

### Camouflaged DENV circumvents antibody binding

**Fig. 3:**
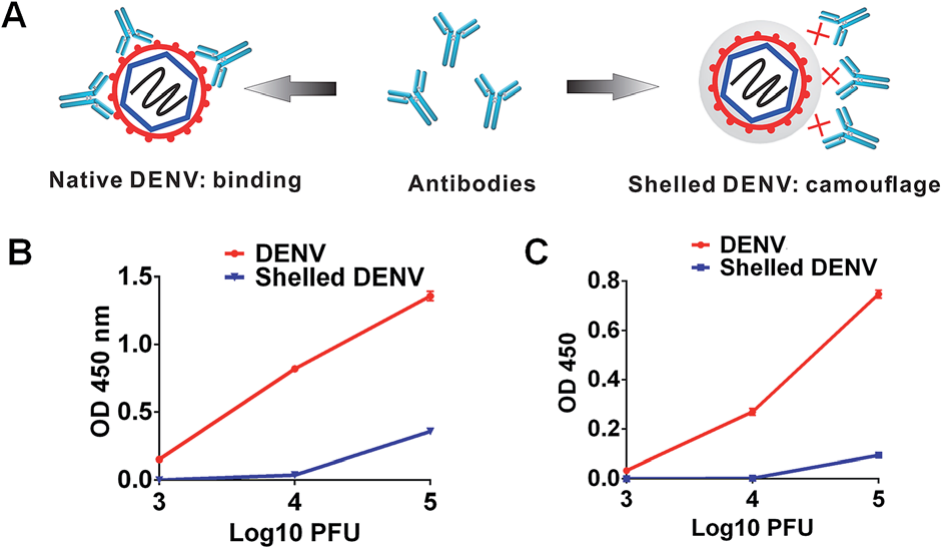
The CaP nanoshell evades antibody recognition. (A) Schematic illustration showing that CaP camouflage inhibits the antibody recognition under extracellular conditions. (B) Binding affinity between 4G2 and the virus (DENV or shelled DENV) ranging from 3 to 5 log 10 PFU. (C) Binding affinity between 2A10G6 and the virus (DENV or shelled DENV) ranging from 3 to 5 log 10 PFU.

Under extracellular conditions, we evaluated the camouflage effect of the CaP shell to inhibit the interaction between the anti-DENV antibodies and DENV. The binding affinities between the shelled DENV and different kinds of antibodies were examined by indirect enzyme-linked immunosorbent assays (ELISAs). Expectedly, all serotypes of bare DENV showed high binding affinities towards the anti-flavivirus monoclonal antibodies, including 4G2 and 2A10G6 26 (Fig. 3B, C and S6†). In contrast, these antibodies were insufficient to recognize the shelled DENV as the binding affinities were substantially reduced (Fig. 3B, C and S6†). In addition, the cross-reactive polyclonal human anti-sera were also incapable of recognizing shelled DENV using a native dot-blot assay (Fig. S7†). As a consequence of insufficient formation of the virus–antibody complex, the CaP shell significantly ablated the ligation of DENV to the Fcγ receptor (FcγR) on K562 cells (Fig. S8†). The CaP shell protected the virus from the antibody recognition and receptor ligation (Fig. 3A), showing its camouflage effect under extracellular conditions.

### Camouflaged DENV abrogates ADE infection *in vitro*

**Fig. 4:**
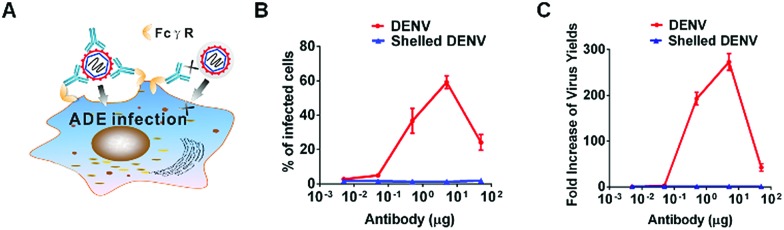
A CaP nanoshell abrogates the ADE of infection *in vitro*. (A) Schematic illustration showing that CaP camouflage inhibits antibody recognition and ablates the FcγR dependent virus infection. (B) DENV or shelled DENV that was first opsonized with serial dilutions of 4G2 was then incubated with K562 cells. The number of DENV-infected cells was measured through flow cytometry. (C) Antibody-enhanced viral production was examined by plaque formation assay. The results were presented as the fold-increase of virus titer after being opsonized with the pre-existing 4G2 antibody.

The camouflage effect of the resulting CaP shell can eliminate the ADE of infection (Fig. 4A). DENV or shelled DENV was opsonized with a serial concentration of a cross-reactive 4G2 antibody to form the virus–antibody complex, which was then allowed to infect K562 cells bearing FcγR. As a result, the intracellular viral load in groups of the native DENV was obviously enhanced by the pre-existing antibody, whilst the shelled DENV completely ablated the enhancement of viral load (Fig. 4B and S9†). The CaP shell abrogated the augment of the intracellular DENV at all tested concentrations of antibody due to its camouflage effect against pre-existing antibodies (Fig. 4B and S9†). This result was further confirmed by evaluating the extracellular virion production under the same conditions. The sub-neutralizing pre-existing antibody induced a 30–300 fold enhancement of the DENV production, whereas the shelled DENV completely abolished the antibody-enhanced virion proliferation (Fig. 4C and S9†). This phenomenon indicated that the superficial CaP directly inhibits the formation of the virus–antibody complex and consequently reduces its ligation to K562, resulting in the circumvention of the ADE of infection (Fig. 4A).

### Camouflaged DENV abrogates ADE of infection *in vivo*

**Fig. 5:**
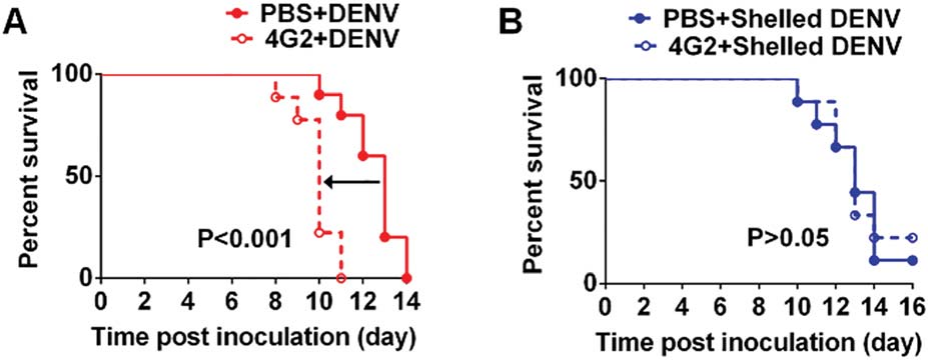
A CaP nanoshell abrogates the ADE of infection *in vivo*. (A) The *in vivo* ADE model revealed that pre-existing 4G2 enhanced DENV infection in mice brains. (B) Shelled DENV abrogated antibody-enhanced DENV infection *in vivo*. Kaplan–Meier survival curves showed the percentage of mice surviving after intracranial infection with DENV or shelled DENV. The differences in survival between the PBS and 4G2 groups were analysed using the log-rank test.

We next investigated whether the CaP shell benefits the abrogation of ADE *in vivo* by using 1 day-old suckling mice. The pre-existing antibody in mice was obtained through the passive transfer of cross-reactive 4G2 monoclonal antibodies, which have been proved to be ADE inductive in mice.^27^ Twenty-four hours after intraperitoneal injection (i.p.) with 4G2 or PBS, the mice were challenged intracranially (i.c.) with the shelled DENV or native DENV. The pre-existing antibody significantly enhanced the disease severity by decreasing the average survival time (AST) from 13 days to 10 days as compared with the control group, representing an enhancement of neurological infection (Fig. 5A, S10 and Table S1†). In contrast, in the shelled DENV group, the mice in control group and ADE group both showed identical AST, verifying that the pre-existing antibody failed to enhance the infection of shelled DENV (Fig. 5B, S10 and Table S1†). Additionally, the shelled DENV recipient induced no extra incidence of death as evidenced by the survival rate, ensuring the safety of the CaP nanoshell (Table S1†). These findings demonstrated the feasibility of biomimetic camouflage to evade ADE *in vivo*.

In our experiment, the ADE of infection manifested enhanced neurovirulence and reduced the average survival time, which is in accordance with the previous report.^28^ However, CaP camouflage blocked the antibody binding and thereby reduced the FcγR-mediated infection in mice brains, resulting in the elimination of early death. Similar strategies from previous observations suggested that the interference with the interplay between DENV and the antibody was capable of abrogating *in vivo* ADE.^27^

### Camouflaged vaccine ablates ADE of infection and preserves immunogenicity

**Fig. 6:**
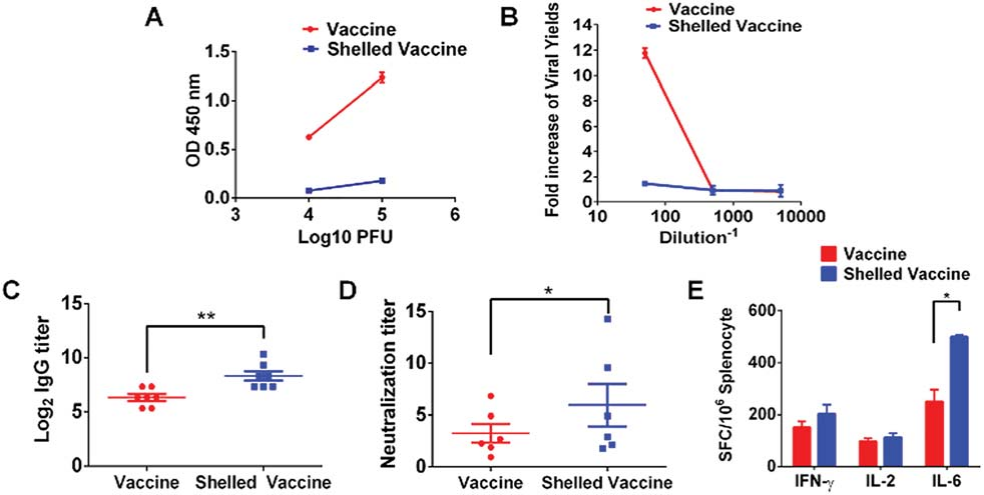
The CaP camouflage abrogates the ADE of infection of the DENV vaccine and improves vaccine efficacy. (A) The CaP shell reduced the binding affinity between the vaccine and anti-sera. (B) Even the attenuated DENV vaccine still faced the ADE of infection in the presence of anti-sera recovered from DENV4-infected Rhesus monkeys, indicating a safety issue with DENV vaccine administration for people with pre-existing antibodies. In contrast, shelled vaccine ablated the ADE of infection. (C) IgG titers and (D) plaque reduction neutralization tests were conducted after immunization with the vaccine or shelled vaccine. (E) ELISPOT assays of IFN-γ, IL-2 and IL-6 secretion were performed using the splenocytes from immunized mice. The data are reported as spot-forming cells (SFC) per 10^6^ splenocytes. The error bars are presented as SDs (Student’s *t* test, *P < 0.05 and **P < 0.01).

ADE of infection has been regarded as one of the biggest challenges in DENV vaccine development. We postulate that such a switchable camouflage would be promising for DENV vaccine engineering. As is well known, ADE occurs when there is co-incidence of sub-neutralizing antibodies and DENV, in spite of a virulent DENV or an attenuated DENV vaccine.^10^,^29^ Thus, the administration of a live attenuated DENV vaccine to individuals, who have a waning antibody response due to a previous DENV infection or vaccination, may place the vaccine recipients at risk of enhanced infection.^28^ To improve the safety of the DENV vaccine, recommendations have been made to restrict the use of the vaccine to those who are not likely to have had prior exposure to DENV.^30^ We hereby examine the possibility of CaP camouflage to evade the ADE of ChinDENV2 infection, a well-characterized live-attenuated chimeric dengue vaccine candidate.^31^ As shown, the presence of anti-sera recovered from DENV-infected rhesus monkeys promoted the ADE of infection of the vaccine, even though the vaccine was highly attenuated (Fig. 6B). This result highlighted a potential safety issue with the DENV vaccine. In contrast, the CaP camouflaged vaccine restricted the recognition of antibodies, resulting in the efficient evasion of the ADE of infection (Fig. 6A and B). Furthermore, to examine the influence of the CaP shell on vaccine efficacy, the mice were injected subcutaneously with the shelled vaccine. As expected, the administration of the shelled vaccine demonstrated enhanced DENV2-specific IgG responses (Fig. 6C) and DENV2-specific neutralizing activities (Fig. 6D). The cytokine-secreting cellular immune responses were also increased compared with those of the native vaccine (Fig. 6E). Moreover, sera collected from vaccine and shelled-vaccine administrated mice did not contribute to ADE induction (Fig. S11†). These findings revealed that the stealth CaP shell not only avoids the ADE of infection but also enhances the efficacy of the vaccine. To test if the CaP nanoshell can influence the adaptive immune responses of the vaccine, we evaluated the ability of CaP nanoparticles to stimulate the cytokine secretion of antigen presenting cells. The RAW264.7 cell line, a macrophage derived cell belonging to antigen presenting cells, is incubated with PBS or pure CaP nanoparticles for 1 hour. After stimulation, CaP enhances the secretion of cytokine TNF-α as compared with the control, indicating that CaP can enhance the activation of immune cells. Therefore, the CaP shell could interact with the immune cells and stimulate the immune process (Fig. S12†). Together, these results verified the improved safety and potency for vaccine administration.

The balance between the induction of protective immunity and the induction of ADE should be of serious consideration in DENV vaccine development. The attenuated vaccine is the only optimal strategy that could be effective and relatively safe. On one hand, the progeny attenuated vaccine would not have the opportunity to encounter antibodies induced by themselves, because the live attenuated vaccine only replicates in limited cycles in cells and is rapidly cleared by the immune system.^31^ On the other hand, when vaccinated people are subsequently exposed to epidemic DENV, the chance of ADE induction is dependent on the efficacy of vaccine. Either neutralization or enhanced virus infection is determined by the threshold concentration of dengue antibodies. When the antibody concentration remains above the neutralization threshold, it will lead to a strong neutralization capability but not ADE infection (Fig. S9B†). Previous reports have demonstrated that the antibodies with strong and long-lasting neutralization capability can minimize the risk of the ADE of infection.^32^

However, scientists are still facing hurdles to developing effective vaccines that can safely protect populations due to the poor understanding of DENV immunity.^33^ Even the most successful vaccine, Dengvaxia (Sanofi Pasteur), still elicits an incomplete immune response to DENV that contributes to the immune enhancement of disease in certain populations.^11^–^13^ The question of whether the dengue vaccine could be safely administered to pre-infected individuals remains unresolved. The development of methods to mask epitopes that may evade “unwanted” interactions is proposed in the development of a respiratory syncytial virus (RSV) vaccine, which also faces the problem of ADE of infection.^34^ But the challenge is to apply the masking strategy to solve the dengue vaccine conundrum. We for the first time propose the use of chemistry based engineering to evade the “unwanted” interactions, by introducing bioinspired and biodegradeable CaP nano camouflage on virus surfaces. The tunable characteristics of CaP nanoshells can cover the virus epitope and preserve its activity and immunogenicity. The camouflage strategy is a proof-of-concept that improves the safety of vaccinations in individuals with pre-existing or waning antibodies.

## Experimental section

### Virus-templated biomineralization

Four serotypes of DENV nanoparticles and ChinDENV2 (diluted to 10^6^ PFU per mL) in 500 μL of serum-free DMEM buffer were incubated with 10 mM calcium chloride (CaCl_2_) at 37 °C for 2 h to induce the site-specific nucleation of calcium phosphate (CaP) on the viral surface. The complex designated as shelled DENV was collected by centrifugation at 8000 *g*.

### Physiochemical characterization

The morphology of shelled DENV nanoparticles was investigated using TEM (JEM-1400, JEOL, Tokyo, Japan) with or without staining. Briefly, the shelled DENV was drop-cast from dispersions onto carbon-coated TEM grids and the solvent was blown away to keep it dry. For negative staining, native virus and shelled DENV were cast onto a carbon-coated TEM grid and stained with phosphotungstic acid before observation. Energy-dispersive X-ray spectroscopy (EDS) analysis of shelled DENV and the CaP control were conducted using TEM.

### Biological characterization

Expression of the viral envelope (E) protein was explored by indirect immunofluorescence assays (IFAs). The confluent BHK-21 cells grown on a glass slide were infected with viruses at a multiplicity of infection (MOI) of 0.01. At 72 h post infection, cells seeded on the coverslip were fixed with an ice-cold acetone/methanol mixture with a ratio of 7 to 3. After drying at room temperature, fixed cells were incubated with cross-reactive monoclonal antibody 2A10G6 to detect the DENV E protein. Cells were washed three times with phosphate-buffered saline (PBS) and then incubated with secondary antibodies conjugated to Alexa Fluor 488 (Invitrogen) in PBS for 30 min at 37 °C. Fluorescent cells were examined using a fluorescence microscope (Olympus).

For the plaque formation assay, BHK-21 cells were used to determine infectivity and viral titer. The samples were incubated with confluent cells in the 12-well plate. The virus containing suspensions were absorbed for 1 h and the cell monolayer was overlaid with DMEM containing 2% FBS and low melting temperature agarose. The cells were incubated for 3 days and overlaid with 4% formalin and crystal violet. The plaques were counted to calculate the number of PFU per mL.

### *In vitro* ADE assay

The ADE assay was performed using K562 cells. Briefly, for the detection of antibody dependent enhancement of DENV infection, DENV or shelled DENV was first pre-incubated with 10-fold serial dilutions of 4G2 under concentrations ranging from 50 to 0.005 μg at 37 °C in 5% CO_2_, allowing the formation of the immune complex. The obtained complex was placed into separated tubes further inoculated with 2 × 10^5^ K562 cells at a MOI of 1 for 2 h. Then the inoculated cells were washed 3 times with serum free DMEM to remove excess and unbound immune complexes. The K562 cells were subsequently re-suspended with 2% maintenance DMEM and cultured for an additional 72 h.

We next used a flow cytometry assay to detect the intracellular expression of the DENV E protein. The K562 cells were washed once with PBS and subsequently fixed in 2% paraformaldehyde for 15 minutes at room temperature. Cells were then rinsed with a PBS buffer containing 1% FBS to remove residual fix solution. For staining, cells were permeabilized with 0.1% saponin (Sigma Aldrich) and stained with an Alexa-488-conjugated (Invitrogen) 4G2 that probed the DENV E protein. The cells were washed twice, and the number of E-protein positive cells was monitored by a flow cytometry instrument (BD Biosciences). The results are expressed as percentage of infected cells *versus* antibody concentration. In a viral yields assay, the inoculated suspension from K562 cells in the ADE assay were collected and then used to conduct a plaque assay on BHK-21 cells.

For the indirect enzyme-linked immunosorbent assays (see ESI†), the DENV-positive human sera were obtained and provided by Guangzhou 8th People’s Hospital. The use of DENV-positive sera was approved by the Ethics Review Committee of Guangzhou 8th People’s Hospital (GPH).

### *In vivo* ADE assessments

All experimental procedures involving animal testing were performed in compliance with the guidelines and protocols approved by the Institutional Animal Care and Use Committee at Beijing Institute of Microbiology and Epidemiology.

The *in vivo* ADE model was established using sucking mice. The enhancement of neurovirulence was observed by the measurement of survival time in the absence or in the presence of 4G2. Groups of 1 day-old sucking mice were injected intraperitoneally with 4G2 or PBS in a dosage of 1 μg per mouse one day before infection. Then, 24 hours after passive injection of antibody, DENV3 was administrated with 20 PFU *via* an intracranial route (i.c.). Animals found in a moribund condition were euthanized and scored as dead within 3 weeks of monitoring. Average survival times (AST) were calculated for animals that succumbed to infection.

### Immunization with shelled vaccines

Immunogenicity was assessed by subcutaneous (s.c.) inoculation with 10^5^ PFU of the ChinDENV2 vaccine or shelled ChinDENV2 into 4 week-old female BALB/c mice. The mice were bled by tail veins after 2 weeks post-immunization. The sera were isolated for the determination of IgG antibodies by an ELISA assay. The neutralizing antibodies against a wild type strain of DENV2 were detected by a 50% plaque-reduction neutralization test (PRNT50). Splenocytes were isolated from the immunized mice at 2 weeks post-immunization and subjected to enzyme-linked immunospot assay.

## Conclusions

We have developed a biomimetic strategy to modify DENV nanoparticles with a CaP camouflage, which is beneficial as it precludes the recognition of different types of pre-existing antibodies, verifying the stealth effect of CaP nanoshells. Both *in vitro* and *in vivo* results have demonstrated that the switchable nature of the CaP shell abrogates the ADE of infection under extracellular conditions and ensures the intracellular immunogenicity, presenting improved administration safety and efficacy. Our results indicate biomimetic, biomineral engineered viruses would be beneficial to reduce the risk of enhanced infection in recipients with a prevalence of sub-neutralizing anti-DENV antibodies, providing a translational camouflage tactic to confer immune evasion and enhance the vaccine potency by using chemical materials.

## Conflicts of interest

The authors declare there is no conflicts of interest regarding the publication of this paper.

## Supplementary Material

Supplementary information
