# Supplementary material for: Biomimetic inorganic camouflage circumvents antibody-dependent enhancement of infection
Source: Chem Sci. 2017 Oct 20;8(12):8240–6. doi: 10.1039/c7sc03868b (PMC5857936; doi:10.1039/c7sc03868b)
Supplement: Supplementary file 1 [file SC-008-C7SC03868B-s001.pdf]

Electronic Supplementary Material (ESI) for Chemical Science.
This journal is © The Royal Society of Chemistry 2017

Electronic Supplementary Information

Biomimetic Inorganic Camouflage Circumvents Antibody-Dependent Enhancement of Infection

Xiaoyu Wang,[a] Yong-Qiang Deng,[b] Dong Yang,[b] Yun Xiao,[a] Hui Zhao,[b] Qing-Gong Nian,[b] Xurong Xu,[a] Xiao-Feng Li,[b] Ruikang Tang*[ac] and Cheng-Feng Qin*[b]

Experimental section

Cells and viruses: BHK-21 cell (a baby hamster kidney cell line) and K562 cell (a human erythroleukemia cell line) were maintained in Dulbecco's minimal essential medium (DMEM; Invitrogen) supplemented with 10% fetal bovine serum (FBS), streptomycin (100 μg/ml) and penicillin (100 U/ml). All cell lines were grown at 37 °C in 5% CO_2. DENV1 strain GZ/80 (GenBank accession No. AF350498), DENV2 strain D2-43 (GenBank accession No. AF204178), DENV3 strain 80-2 (accession No. AF317645), DENV4 strain GZ B5 (GenBank accession No. AF289029), and the chimeric dengue virus vaccine candidate that composed of the prM and the E proteins of DENV2 within the backbone of an attenuated Japanese encephalitis virus (JEV) vaccine (ChinDENV2) were prepared as previously described.[1] The virus titer was determined using a standard plaque assay on BHK-21 cells. The virus stocks were stored in aliquots at −80 °C until further use. All the results in the main text were represented by DENV3, while the correspondence results from the other three serotypes were included in supporting information.

Enzyme-linked immunosorbent assays (ELISA) :

Indirect ELISA: The 96-well enzyme immunoassay Costar plates were first coated with 100 μl of 5 μg/ml 4G2 or 2A10G6 IgG diluted in 50 mM sodium carbonate buffer (pH 9.6) overnight at 4 °C. The coated plates were blocked with a 2% (wt/vol) solution of BSA in PBS at 37 °C for 2 h.

The plates were rinsed with PBST (PBS with 0.05% Tween 20) before use. To each well, 100 µl of DENV or shelled DENV diluted in serum free DMEM was added and reacted at 37 °C for 1.5 h. The viruses captured by 4G2 or 2A10G6 antibody were detected subsequently with clinical anti-DENV serum. The products were then conjugated with horseradish peroxidase for 45 min. The tetramethylbenzidine substrate was added, and the reaction was stopped by addition of 100 µl of 1 N phosphoric acid. Absorption was measured at 450 nm by ELISA plate reader.

Direct ELISA: In the direct ELISA assay, DENV virion suspended in coating buffer was directly coated on the bottom of microtiter plates. The serials dilutions of IgG from collected sera were then added to the DENV embedded plate. The developing of optical colors during ELISA assay indicated that IgG recognize the envelope protein (E) on the surface of DENV virion.

Viral bound assay**:** The cellular attachment of shelled DENV-antibody or DENV-antibody complex to the FcγR on K562 cell line was evaluated. Either DENV or shelled DENV was first opsonized with 4G2 or PBS at 37 °C for 1 h to produce the immune complex. The K562 cells were then exposed to the virus-antibody complex at a MOI (multiplicity of infection) of 5 for 2 h at 4 °C. Cells were then washed three times with cold DMEM containing 2% FCS to remove unbound virus-antibody complex. The number of virus complex that bound to the cells surface was determined by one-step quantitative real-time RT-PCR method. GAPDH (glyceraldehyde-3-phosphate dehydrogenase) was used as an internal gene control in the reactions.

Quantitative reversed real-time PCR (qRT-PCR): Viral RNA samples from suspension or cells pellets were extracted using RNA PurelinkTM RNA mini kit (Ambion) following the manufacturer's specifications. The obtained RNA was quantified by reversed quantitative real-time PCR using One Step PrimeScript® RT-PCR Kit (Takara) according to the manufacturer's

instructions. Primers (DV-F 5' GGAAGGAGAAGGACTGCACA-3' and DV-R 5' ATTCTTGTGTCCCATCCTGCT-3') targeted to the nonstructural region of the DENV capsid gene, which is highly conserved among the four DENV but not in other flaviviruses. Virus titers were calculated from a standard curve generated by serially dilution of standard viral RNA.

Supplementary figures:

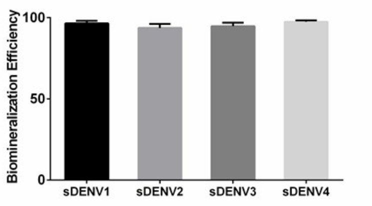

Fig. S1. Biomineralization efficiency (shelled DENV, sDENV). The viral nanoparticles suspended in DMEM are composed of ~30% of immature viral particles containing uncleavaged PrM. The result of mineralization efficiency (above 95%) suggested that the majority of immature DENV particles (~30%) are biomineralized during the treatment.

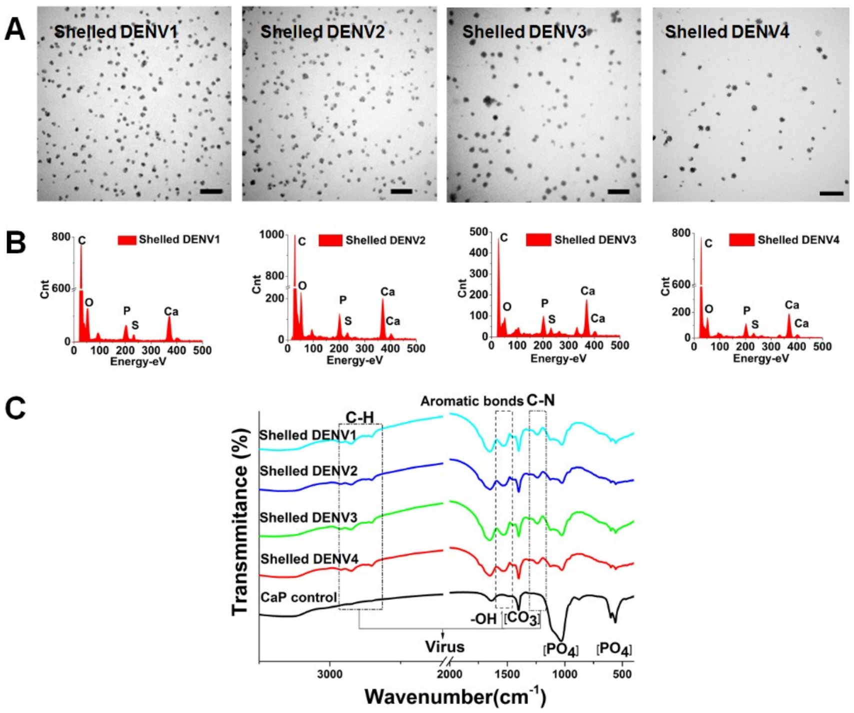

Fig. S2. (A) Non-stained TEM showed the successful biomineralization of DENV with all four serotypes, including shelled DENV1, shelled DENV2, shelled DENV3, and shelled DENV4 (scale bar: 500 nm). (B) EDS investigation of shelled DENV. (C) FT-IR analysis of shelled DENV and CaP control.

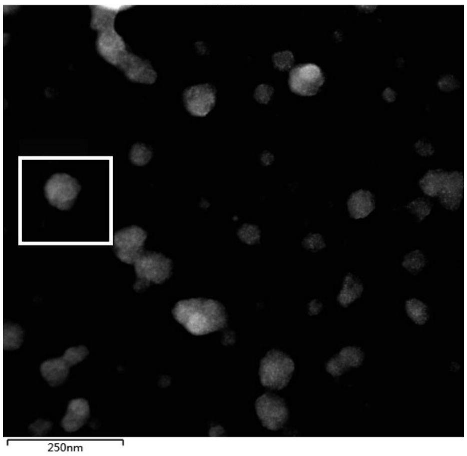

Fig. S3. ROI of EDS elemental mapping using transmission electron microscopy (STEM). The white square indicated the selected shelled DENV.

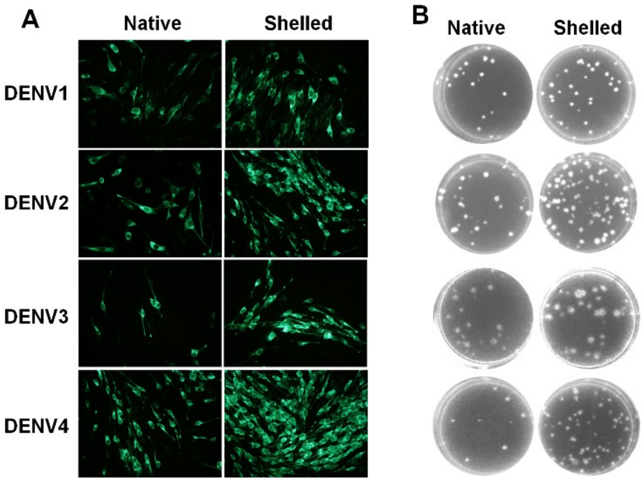

Fig. S4. The CaP nanoshell is reversible to restore original bioactivity. (A) Detection of the viral envelope protein by DENV-specific antibody 2A10G6 after infection with the shelled DENV or DENV using indirect immunofluorescence assay (IFA) at 72 h post infection. (B) The infectivity of native DENV or shelled DENV was examined using an end point plaque formation assay at 72 h post infection.

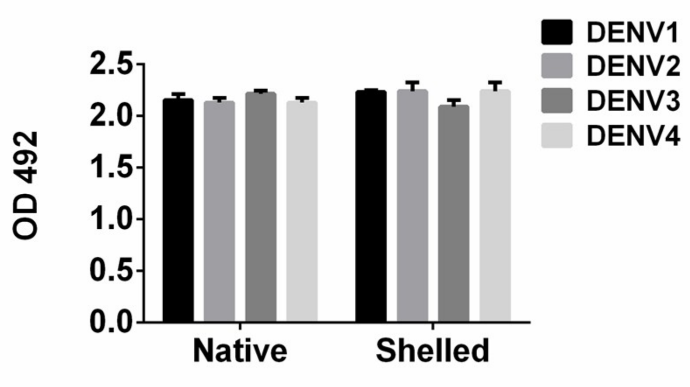

Fig. S5. MTS assay examined the cytotoxicity of native DENV and shelled DENV in K562 cells. CaP shell showed no extra cytotoxicity in K562 cell that is nonpermissive for DENV.

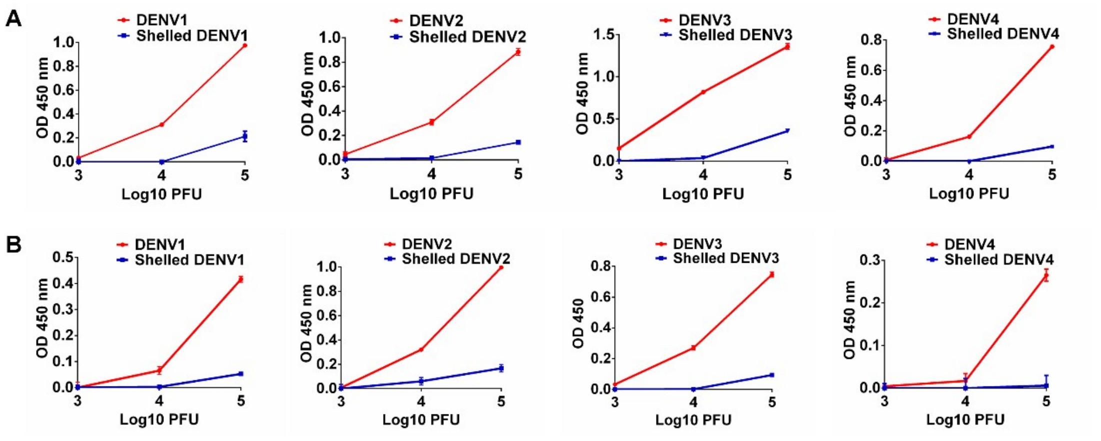

Fig. S6. Binding affinity between (A) 4G2 and virus (DENV or shelled DENV) ranging from 3 to 5 log 10 PFU. (B) Binding affinity between 2A10G6 and virus (DENV or shelled DENV) ranging from 3 to 5 log 10 PFU.

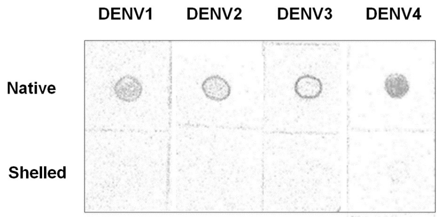

Fig. S7. *In situ* dot blot assay under native conditions using cross-reactive human anti-DENV serum.

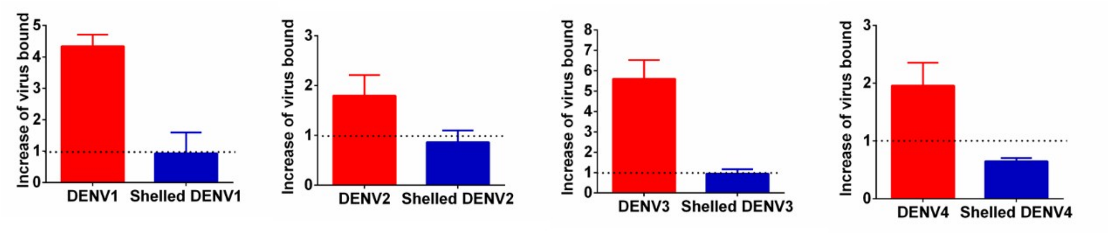

Fig. S8. Cellular bound assay. As known, K562 cell is minimally permissive to native DENV infection. However, under the mediation of Fcγ receptor, the Fc portion of antibody will promote the bound of the DENV-antibody complex, leading to the augment of virus infection. DENV or shelled DENV was incubated with sub-neutralizing antibody to form the virus-antibody complex, which was then allowed to interact with K562 at 4 °C for 2 h. As a result, an approximately 1.5- to 5.5-fold increase of virus attachment was observed in the group of DENV-antibody complex as compared with group treated without antibody. On the contrary, in groups of shelled DENV, the presence of antibody failed to increase the viral bound, indicating a camouflage effect of CaP nanoshell.

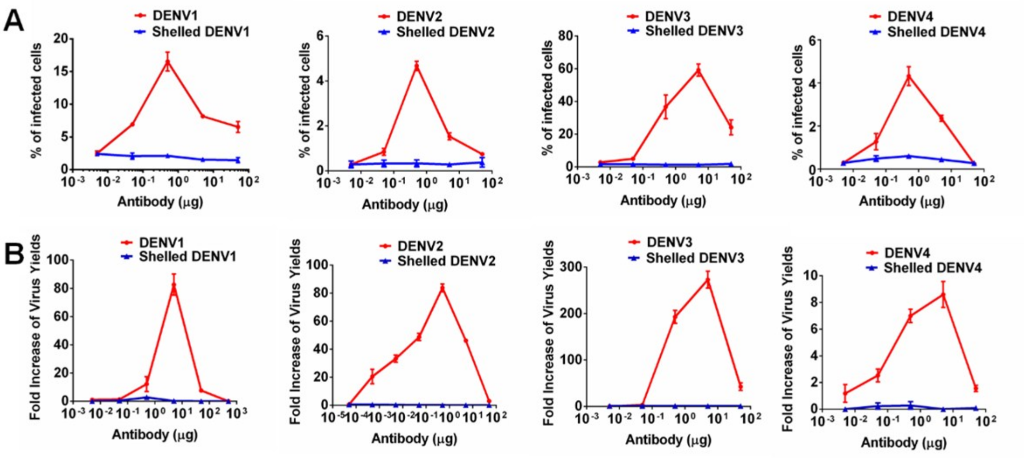

Fig. S9. ADE infection occurs when pre-existing antibodies do not neutralize infection but cross reacted with virus and ligated it to Fcγ receptor on cells, resulting in the promotion of virus entry and virus production. Shelled DENV abrogated the ADE infection *in vitro* due to its camouflage effect. (A) The frequency of DENV-positive cells. (B) The viral yield assay.

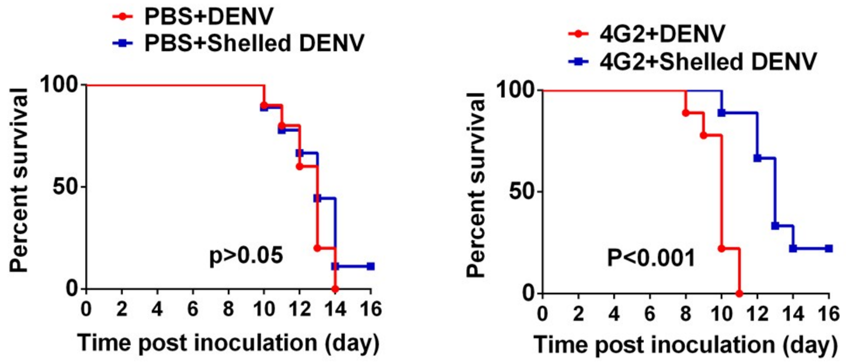

Fig. S10. Kaplan-Meier survival curves. Generally, DENV is unable to replicate well in immunocompetent mice, but is susceptible in the brain of sucking mouse. [2] We use sucking mice as model mice because the i.c. injections of DENV can induce obviously neurological diseases and death. No difference was observed in the survival curves of sucking mice infected with DENV or shelled DENV in the absence of 4G2 (left). In the presence of 4G2 antibody, bare DENV enhanced the severity of infection while shelled DENV abrogated antibody-enhanced DENV infection (right). The differences between DENV and shelled DENV administrated groups were analyzed using the log-rank test.

Table S1. Effect of ADE on mortality and survival.

Virus	Dose (PFU)	Mortality (Dead/Total)	Median survival [a] (day)	p-value vs. PBS+DENV3 control [b]
PBS+DENV3	20	100% (10/10)	13	
4G2+DENV3	20	100% (9/9)	10	*** (P=0.0001)
PBS+shelled DENV3	20	88.9% (8/9)	13	
4G2+shelled DENV3	20	77.8% (7/9)	13	

[a] Mean survival time of mice who succumbed to infection during 21-day time course.

[b] Results of Log-rank (Mantel-Cox) Test comparing 4G2 recipients to PBS recipients at the same viral dose.

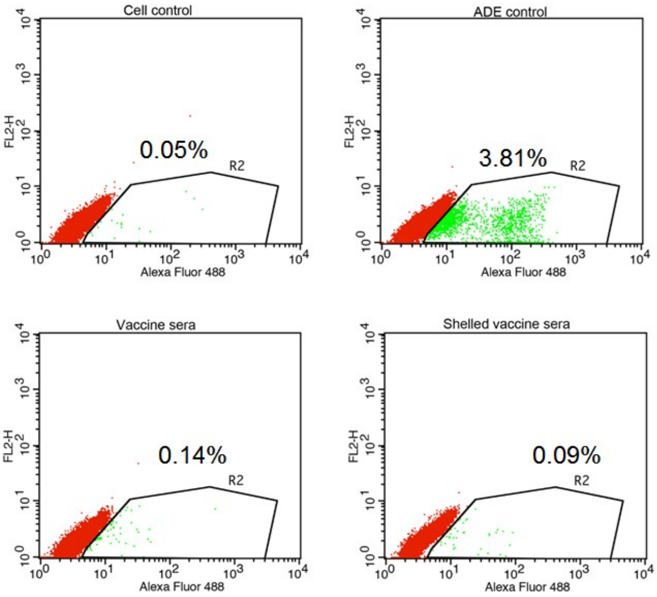

Fig. S11. We collected and mix the sera from groups (5 mice/group) immunized with vaccine and shelled-vaccine for 4 weeks. Sera without any dilution were first pre-incubated with DENV1 to produce immune complexes, which were then incubated with K562 cells for 1 hour. Cells were then washed 3 times and cultured for an additional 72 h. We next used flow cytometry assay to detect intracellular expression of DENV E protein (green).

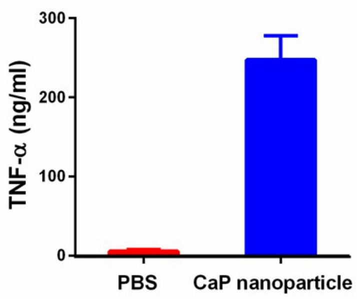

Fig. S12. Secretion of TNF-α by macrophages exposed to CaP nanoparticles for 2h.

References:

[1] X. F. Li, Y. Q. Deng, H. Q. Yang, H. Zhao, T. Jiang, X. D. Yu, S. H. Li, Q. Ye, S. Y. Zhu, H. J. Wang, Y. Zhang, J. Ma, Y. X. Yu, Z. Y. Liu, Y. H. Li, E. D. Qin, P. Y. Shi, C. F. Qin, *J. Virol.* **2013**, *87*, 13694.

[2] A. J. Johnson, J. T. Roehrig, *J. Virol.* **1999**, *73*, 783.
